# qSNE: quadratic rate t-SNE optimizer with automatic parameter tuning for large datasets

**DOI:** 10.1093/bioinformatics/btaa637

**Published:** 2020-07-14

**Authors:** Antti Häkkinen, Juha Koiranen, Julia Casado, Katja Kaipio, Oskari Lehtonen, Eleonora Petrucci, Johanna Hynninen, Sakari Hietanen, Olli Carpén, Luca Pasquini, Mauro Biffoni, Rainer Lehtonen, Sampsa Hautaniemi

**Affiliations:** Research Program in Systems Oncology, Research Programs Unit, Faculty of Medicine, University of Helsinki, 00014 Helsinki, Finland; Research Program in Systems Oncology, Research Programs Unit, Faculty of Medicine, University of Helsinki, 00014 Helsinki, Finland; Research Program in Systems Oncology, Research Programs Unit, Faculty of Medicine, University of Helsinki, 00014 Helsinki, Finland; Research Center for Cancer, Infections and Immunity, Institute of Biomedicine, University of Turku, Turku 20014, Finland; Research Program in Systems Oncology, Research Programs Unit, Faculty of Medicine, University of Helsinki, 00014 Helsinki, Finland; Department of Oncology and Molecular Medicine, Istituto Superiore di Sanità, Rome 00161, Italy; Department of Obstetrics and Gynecology, University of Turku and Turku University Hospital, Turku 20521, Finland; Department of Obstetrics and Gynecology, University of Turku and Turku University Hospital, Turku 20521, Finland; Research Program in Systems Oncology, Research Programs Unit, Faculty of Medicine, University of Helsinki, 00014 Helsinki, Finland; Research Center for Cancer, Infections and Immunity, Institute of Biomedicine, University of Turku, Turku 20014, Finland; Department of Pathology, University of Helsinki and HUSLAB, Helsinki University Hospital, Helsinki 00014, Finland; Major Equipments and Core Facilities, Istituto Superiore di Sanità, Rome 00161, Italy; Department of Oncology and Molecular Medicine, Istituto Superiore di Sanità, Rome 00161, Italy; Research Program in Systems Oncology, Research Programs Unit, Faculty of Medicine, University of Helsinki, 00014 Helsinki, Finland; Research Program in Systems Oncology, Research Programs Unit, Faculty of Medicine, University of Helsinki, 00014 Helsinki, Finland

## Abstract

**Motivation:**

Non-parametric dimensionality reduction techniques, such as t-distributed stochastic neighbor embedding (t-SNE), are the most frequently used methods in the exploratory analysis of single-cell datasets. Current implementations scale poorly to massive datasets and often require downsampling or interpolative approximations, which can leave less-frequent populations undiscovered and much information unexploited.

**Results:**

We implemented a fast t-SNE package, qSNE, which uses a quasi-Newton optimizer, allowing quadratic convergence rate and automatic perplexity (level of detail) optimizer. Our results show that these improvements make qSNE significantly faster than regular t-SNE packages and enables full analysis of large datasets, such as mass cytometry data, without downsampling.

**Availability and implementation:**

Source code and documentation are openly available at https://bitbucket.org/anthakki/qsne/.

**Supplementary information:**

Supplementary data are available at *Bioinformatics* online.

## 1 Introduction

Single-cell measurement technologies have become routinely used tools in medical research (Heath *et al.*, 2016; Shalek and Benson, 2017; Stuart and Satija, 2019). While these technologies offer unprecedented opportunities to understand diseases at a single-cell resolution, the vast quantity and the high dimension of the data pose challenges for the analysis. For example, mass cytometry allows simultaneously quantification of tens of proteins from hundreds of thousands of individual cells (Amir *et al.*, 2013; Levine *et al.*, 2015; Spitzer and Nolan, 2016) and single-cell RNA-seq technology tens of thousands of genes in thousands of cells (Heath *et al.*, 2016; Shalek and Benson, 2017; Stuart and Satija, 2019). As a research project commonly features hundreds of samples, the paucity of analysis tools designed to scale to these dimensions hinders the exploitation of the information in the data to the fullest.

Non-parametric dimensionality reduction techniques, such as t-distributed stochastic neighbor embedding (t-SNE) (Amir *et al.*, 2013; Linderman *et al.*, 2019; van der Maaten and Hinton, 2008) and uniform manifold approximation and projection (UMAP) (Becht *et al.*, 2019; McInnes *et al.*, 2018) are the most frequently used methods in exploratory single-cell data analysis (Becht *et al.*, 2019; Butler *et al.*, 2018; Cao *et al.*, 2019; Levine *et al.*, 2015; Tasic *et al.*, 2018). Despite being derived from different assumptions, in fact, the methods are very similar in nature and the differences can be attributed to hyperparameter choices and approximation schemes (McInnes *et al.*, 2018, see Supplementary Material). While t-SNE seems to retain clusters qualitatively better, UMAP tends to be better on continuous trajectories in practice (Becht *et al.*, 2019), but this has been suggested to be solely due to different initialization (Kobak and Linderman, 2019). Currently, t-SNE is the most commonly used method, especially in the mass cytometry field (Amir *et al.*, 2013; Becht *et al.*, 2019; Levine *et al.*, 2015; Spitzer and Nolan, 2016).

The main issue with the standard t-SNE is that the optimization process is naive (gradient descent) and slow. To counter this, downsampling has been traditionally used (Amir *et al.*, 2013; Bendall *et al.*, 2012; Qiu *et al.*, 2011) and, more recently, interpolation schemes have been proposed (Gisbrecht *et al.*, 2015; Linderman *et al.*, 2019; van der Maaten, 2014). However, these strategies remain problematic, as less-frequent populations are likely filtered out or get intermixed in the larger patterns (as interpolation omits high-frequency features, and thus information). This can be a problem, as e.g. even a small malignant population can give rise to cancer progression due to evolutionary pressure (Agarwal and Kaye, 2003; Heath *et al.*, 2016; Shaffer *et al.*, 2017). Further, the algorithm is sensitive to the selection of a fixed perplexity (a scale or level of detail parameter), which necessitates parameter tuning from the data analyst. Combined with poor performance on large datasets (Belkina *et al.*, 2019; Linderman *et al.*, 2019), this makes the whole process of analyzing the data laborious. Finally, the original t-SNE algorithm makes no attempt to evaluate how faithfully the visualization represents the underlying data.

To address these issues, we implemented (i) a solver that converges methodologically faster and requires no tuning of the gradient descent parameters; (ii) an automatic parameter selection process, which removes the need for manual perplexity tuning; and (iii) a quality metric, which can be used to assess whether the projected model captures the original high-dimensional data. Our improvements are complementary and can be combined with previous efforts (Belkina *et al.*, 2019; Gisbrecht *et al.*, 2015; Linderman *et al.*, 2019; van der Maaten, 2014), and they are general enough to be combined with GPU acceleration schemes (Chan *et al.*, 2019; Pezzotti *et al.*, 2020). We show that the improvements alone enable full analysis of large mass cytometry datasets, which reveals novel phenotypic structures not visible in the downsampled data. Our implementation, qSNE, is available at https://bitbucket.org/anthakki/qsne/ under an open (BSD) license.

## 2 Materials and methods

### 2.1 The t-SNE algorithm

The t-distributed stochastic neighbor embedding (t-SNE) finds a lower-dimensional representation of a dataset such that the distribution of local distances between the samples is maintained (van der Maaten and Hinton, 2008). More specifically, it optimizes the information lost (Kullback–Leibler divergence) when using a low-dimensional distribution *Q* to approximate the high-dimensional neighbor distribution *P*:

(1) $$C≐D_{\;\text{KL}}(P\;||\;Q)=\sum_{i=1}^{m}\sum_{j=1}^{m}-p_{ij}\;\;\text{log}\;\frac{q_{ij}}{p_{ij}}$$

where *m* is the number of samples and *p_ij_* (*q_ij_*) are the high (low)-dimensional densities of the distribution *P* (*Q*) between the samples *i* and *j*. t-SNE uses a normal distribution for *P* and a t-distribution for *Q* (van der Maaten and Hinton, 2008; see Supplementary Material for details), but other distributions are possible, the normal distribution representing a diffusive random walk between the samples (see Supplementary Material). The diffusivity of the input space *P* is controlled by the standard deviation *σ_i_* of the normal distribution around the sample *i*, which is set by a global perplexity parameter *π* representing the number of relevant neighbors (van der Maaten and Hinton, 2008). The original algorithm by van der Maaten and Hinton (2008) uses gradient descent and a momentum term to optimize the intricate cost function.

### 2.2 The L-BFGS algorithm

A gradient descent scheme only allows linear convergence, which can be prohibitively slow on large datasets. Quadratic methods (such as Newton’s method) permit quadratic convergence, but evaluating the Hessian matrix directly is too expensive, so we use the limited-memory Broyden–Fletcher–Goldfarb–Shanno method (L-BFGS) (Liu and Nocedal, 1989), which uses rank-1 updates inferred from the previous updates and their gradients to numerically estimate a Newton search vector [see Supplementary Material, Algorithm (SA1)]. This combines potentially quadratic convergence with low computational overhead as the full Hessian matrix need not to be evaluated, but a low-rank approximation is used, and even that need not to be explicitly formed in the memory. Provided that the low-rank approximation can retain most of the power of the true Hessian matrix, the performance remains comparable to a true Newton method. A Newton method is also in advantageous in the sense that the step size is naturally set by the Hessian matrix magnitude.

### 2.3 Automatic perplexity selection

The neighborhood entropy $H(P_{i})$ is a monotonic increasing curve from 0 to log ⁡(m−1) as the bandwidth *σ_i_* varies from 0 to *∞*. This entropy curve is used to locate the bandwidth corresponding to the specified perplexity value *π*. However, the curve can be also exploited to identify the bandwidths where the neighborhood structure remains insensitive. This holds also in the presence of multiple local scales, as a scale only contributes to the entropy gradient at the sensitive regions. In practice, this results in a staircase-like figure (see e.g. Fig. 3) where flat regions correspond to uninteresting perplexity values and highly transient sensitive. Given a perplexity range, the optimum can be located using sectioning [see Supplementary Material, Algorithm (SA3)]. We denote the optimized bandwidths by $\sigma_{i}^{*}$ and the corresponding perplexity values $\pi_{i}^{*}$, and the latter no longer need to be fixed over the dataset, which also allows different (optimal) perplexity at different regions of the space.

### 2.4 Quality of an acquired mapping

For any mapping in the t-SNE framework, the source entropy *H*(*P*) represents the average number of bits needed to encode a sample of the original neighbor relationship, while the Kullback–Leibler divergence between the source and destination distributions $D_{\;\text{KL}}(P\;||\;Q)$ is the average number of extra bits needed if the output model is used encode the samples instead. These are readily available during the optimization, and can be evaluated once the optimal mapping has been obtained.

To quantify the quality of the mapping, we propose the following normalized statistic:

(2) $$q≐1-\frac{\sum_{i=1}^{m}H(P_{i}^{*})}{\sum_{i=1}^{m}H(P_{i}^{*})+D_{\;\text{KL}}(P_{i}^{*}\;||\;{\hat{Q}}_{i})}$$

where $P_{i}^{*}$ is the distribution around the *i*th sample for its optimal bandwidth and ${\hat{Q}}_{i}$ is the optimal embedding distribution. This quantity has the following rationale: $H(P^{*})$ quantifies the bits needed to represent the samples in the original space, while the cross-entropy $H(P^{*})+D_{\;\text{KL}}(P^{*}\;||\;\hat{Q})$ represents the number of bits needed to encode the data using the low-dimensional model, their ratio being the fraction of samples encoded in the same space with the output model. As expected, *q* is zero for one-to-one correspondence between the source and destination distributions ($D_{\text{KL}}=0$), and unity if all the information is lost ($D_{\text{KL}}=\infty$). In practice, $H(P_{i}^{*})$ is obtained as a side product of the automatic perplexity selection [through Equation (S5) after Algorithm (SA3)] and $D_{\;\text{KL}}(P_{i}^{*}\;||\;{\hat{Q}}_{i})$ as a side product of obtaining the t-SNE mapping for the optimized bandwidths [through Equation (S4) after Algorithm (SA4)].

### 2.5 Datasets used for evaluation

To illustrate the advantages of our method, qSNE, we used two publicly available (human bone marrow and MNIST) and one unpublished high-grade ovarian cancer (HGSOC) dataset. The advantage of the bone marrow and MNIST datasets is that they are manually labeled and thus it is possible to quantify whether the visualization is meaningful. For the purposes of comparison, the datasets were downsampled as it is not practical to run the original t-SNE algorithm on 100 000 s of samples, especially with various parameters. Meanwhile, the HGSOC dataset demonstrates that the improvements in qSNE enable discovering novel biomedical insights from cancer patient samples. We also analyzed a Splatter generated (Zappia *et al.*, 2017) single-cell RNA-seq dataset in the Supplementary Material, which features a much higher dimension (18 726 genes).

The first dataset, available at https://github.com/lmweber/benchmark-data-Levine-32-dim, quantifies a panel of surface protein markers for single cells from human bone marrow profiled using time-of-flight mass cytometry (CyTOF) measurements, which were originally used to study phenotypic heterogeneity of acute myeloid leukemia (AML) patients (Levine *et al.*, 2015). The data features a total of 104 184 manually gated (labeled) cells with 32 protein markers from two individuals, and represents a typical experimental setting for a CyTOF measurement.

Second, we use the MNIST database (Lecun *et al.*, 1998), available at http://yann.lecun.com/exdb/mnist/, which is a collection of handwritten digits (from 0 to 9). We only used the training set part of the dataset, featuring 60 000 labeled samples, which are 28 × 28 pixel images of 256 gray levels each (regarded as 784-dimensional vectors). This dataset has been frequently used to benchmark machine learning methods, and was used for evaluation e.g. by van der Maaten and Hinton (2008).

The third dataset consists of CyTOF measurements of ascites samples harvested from a single HGSOC patient, before and after administering chemotherapy. These data contains 27 surface protein markers in 98 512 single cells in the primary (before chemotherapy) and 127 874 cells in the interval (after chemotherapy) sample.

## 3 Results and discussion

### 3.1 Faster t-SNE mapping through quasi-Newton optimization

We implemented a quasi-Newton optimizer, based on the limited-memory Broyden–Fletcher–Goldfarb–Shanno (L-BFGS) algorithm (Liu and Nocedal, 1989), on the t-distributed stochastic neighbor embedding (t-SNE) objective, which permits quadratic convergence (Liu and Nocedal, 1989) as opposed to the linear convergence of the gradient descent used in the original implementation (van der Maaten and Hinton, 2008). The L-BFGS optimizer exploits a numerical estimate of the local curvature to allow converge in ∼30 iterations ($\sqrt{1,000}$) versus the 1000 of the original variant, which yields an order of magnitude speedup even on modestly sized datasets.

To evaluate the performance of qSNE, we used a human bone marrow mass cytometry dataset (Levine) (Levine *et al.*, 2015), which well represents the experimental setting of a mass cytometry measurement and has also been manually gated, which provides ground truth for performance evaluation (Levine *et al.*, 2015). The corresponding results for the MNIST dataset (Lecun *et al.*, 1998) are shown in Supplementary Figures S1 and S2, and Splatter-generated (Zappia *et al.*, 2017) single-cell RNA-seq data in Supplementary Figures S5 and S6.

**Fig. 1.**
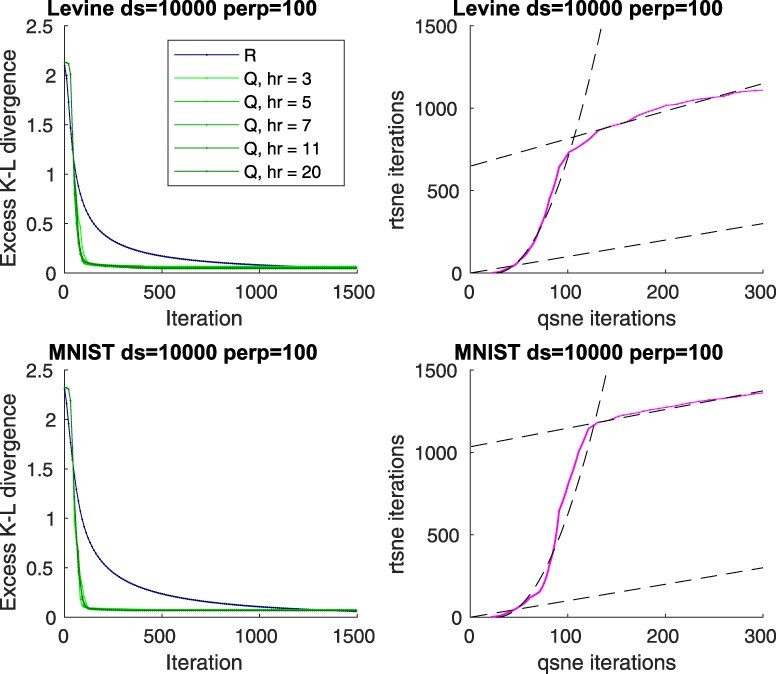
Convergence of qSNE versus a linear t-SNE implementation. Left panels: Progress, as quantified by the excess objective value above the optimum (determined experimentally) as a function of number of iterations for the Levine (Levine *et al.*, 2015) and MNIST (Lecun *et al.*, 1998) datasets, randomly downsampled to 10 000 samples at perplexity 100, for both our quadratic implementation (Q) with various ranks of Hessian matrix approximation (hr) and for a linear Rtsne implementation (R). Right panels: Number of consumed iterations for equal progress (objective value) for the two methods (magenta curve). The dashed black lines indicate a linear or a quadratic fit. (Color version of this figure is available at *Bioinformatics* online.)

To verify that the L-BFGS optimizer operates in the quadratic converge region in a typical setting, we compared how the t-SNE objective—Kullback–Leibler (K–L) divergence between distributions of the pairwise distances of the points in the input and output space—evolves as a function of the iteration count. The results in Figure 1 suggest that in the initial region the L-BFGS optimizer attains a superlinear convergence, while no such effect can be observed with a gradient descent optimizer, as expected. By comparing the iteration counts required for equal progress, as shown in Figure 1, we verified that the convergence of the L-BFGS optimizer is indeed quadratic with respect to that of the gradient descent in the beginning of the optimization. We report that typically the convergence of the L-BFGS optimizer is quadratic in the beginning, but as the optimizer quickly arrives near to the optimum, the rate drops to linear as precision starts to limit the process. Still, the linear rate of convergence remains faster with the L-BFGS optimizer, likely as the learning rate is optimized rather than fixed. We also note that there is a natural warm-up of few iterations, as the L-BFGS optimizer needs to collect curvature information before quadratic speed can be attained.

The results show also that the exact rank of the Hessian matrix approximation plays a minor role in the qualitative behavior of the convergence on these data. While a larger rank generally allows faster convergence, even constant-rank approximations feature the benefit of quadratic convergence and only incur a constant overhead per iteration, suggesting that the strategy is viable for speeding up practical large-scale problems. This is of course only possible if the data are inherently locally, but not necessarily globally, low-dimensional but embedded in a higher-dimensional space, which often is the case and can be expected, for example, in gene expression datasets due to inherent correlations.

**Fig. 2.**
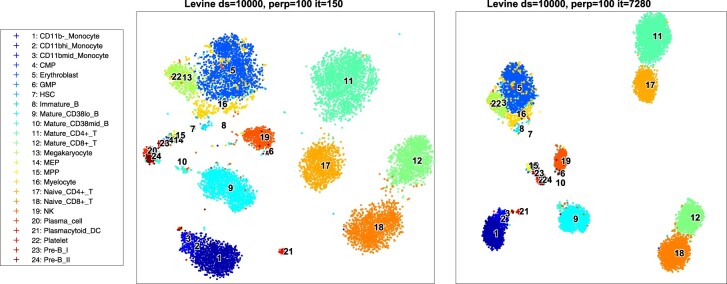
Levine bone marrow data mapped into 2-D. Left panel: qSNE with rank-11 Hessian matrix approximation after only 150 quasi-Newton iterations; and right panel: t-SNE after 7280 iterations (cf. Fig. 1). The datasets were randomly downsampled to 10 000 samples and perplexity is set to 100 in both cases

Often the cost surface features multiple local optima, which may imply convergence to a different optimum for different optimization paths. To evaluate whether the obtained visualizations are useful after the short quadratic walk, we visualized the projections for the two methods. The results are illustrated in Figure 2 and Supplementary Figure S1. The obtained projections appear qualitatively similar: the bone marrow data captures the hematopoietic developmental lineages in both the qSNE and Rtsne mappings (Levine *et al.*, 2015). Specifically, the hematopoietic stem cells and progenitors map in the center of the projection, while the more differentiated and matured cells are located at the exterior of the plot, and mature T-cells map to the furthest distance from the center. As shown in Supplementary Figure S2, the conclusions regarding the convergence and quality of mappings hold for various datasets, downsampling factors, and for various perplexity and optimization parameters.

In terms of consumed CPU time qSNE is much faster than Rtsne (v0.15, using van der Maaten’s C++ implementation, see Supplementary Material), as shown in Supplementary Figure S3. With a single thread, an analysis for qSNE takes ∼15 min to 2 h, while the same analyses for Rtsne take ∼2–25 h. The main benefit comes from the fact that qSNE requires an order of magnitude fewer iterations for convergence, but on the other hand the cost per iteration is slightly larger (by a constant factor if the Hessian matrix rank is O(1)), but small enough to give a distinct benefit and to be insignificant even when considering an equal number of iterations. Furthermore, qSNE can fully utilize parallelization at both vector instruction and thread levels, which can give yet another order of magnitude of advantage for practical analyses in terms of wall clock time.

### 3.2 Automatic bandwidth selection reduces parameter tuning

The choice of the perplexity parameter can have big impact to the resulting t-SNE mapping and finding an optimal value through trial and error is tedious. We show that most perplexity values are not very interesting, exerting very little changes on the acquired mapping, and such regions can be automatically detected (detailed in the Section 2). This allows the analyst to focus on the relevant perplexity values. On the other hand, the perplexity parameter can be fine-tuned to an optimal value around a chosen bandwidth, provided that a sufficiently narrow range with a single optimum is selected. Moreover, a variable bandwidth across the sample space can be advantageous in case the space is not uniform, but contains clusters with various bandwidths.

**Fig. 3.**
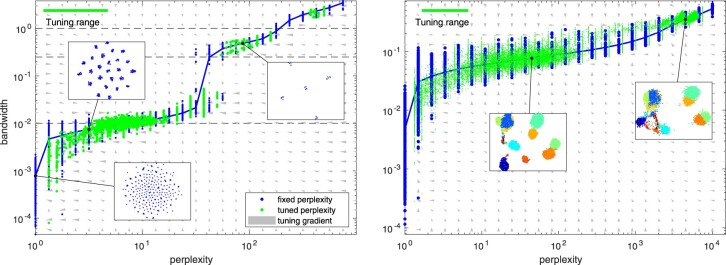
Perplexity versus bandwidth with fixed and automatic perplexity selection. Left panel: an artificial dataset of 10-D hierarchically normal data with five clusters with five subclusters each. The blue dots visualize the effective bandwidth (the chosen *σ_i_* parameter) for each sample with the blue curve showing their median. Meanwhile, the green dots visualize the effective perplexity versus the effective bandwidth for each sample when the perplexity is automatically tuned by a factor of [2^–1^, 2]. The gray arrows indicate the estimated gradient field for the perplexity tuning process. The dashed black lines indicate the true bandwidths in the generated data. Visualizations of the resulting mappings are shown in insets. Right panel: the corresponding plot for Levine data downsampled to 15 000 samples. (Color version of this figure is available at *Bioinformatics* online.)

To exemplify the operation of the automatic perplexity selection, we generated a 10-dimensional synthetic dataset with five clusters [with standard deviation (SD) of 1] each having five subclusters (with SD of 0.25) with a total of 1000 samples (with SD of 0.01). Depending on the selected perplexity range, qSNE results in one of the possible representation of the dataset: the least perplexity optimum corresponds to the setting where each sample is separated into a separate cluster; the next one reveals each of the 25 subclusters; and the third the five high-level clusters, as shown in Figure 3. The highest perplexity setting will opt to form a single cluster for all the data points. The question which one of these is the most useful is of course up to the data analyst, but the number of embeddings with differing structure can be analyzed in the perplexity-bandwidth plot and fine tuning of the perplexity value can be automatically performed and allows discovering this hierarchy.

To demonstrate that the automated perplexity tuning is useful in practice, we performed perplexity analysis on the Levine dataset. For these data, the interesting levels of detail regarding a 2-D projection correspond to (i) 3-clustering into B-cells, T-cells and in less tissue-specific cells; or (ii) into a more detailed clustering including hematopoietic stem and progenitor cells and their differentiated forms (as shown in Fig. 2). Specifically, for the dataset downsampled to 15 000 points, these two clusterings are attracted roughly from the perplexity regions [20, 100] and [2000, 10 000]. The perplexity-bandwidth plot for the Levine dataset is shown in Figure 3, where the insets indicate the optimal projections.

### 3.3 Information lost in the t-SNE mappings

A projection from a high-dimensional dataset into lowerdimension loses information, so it is useful to evaluate how well the projection represents the original data. For this, we suggest to compute the fraction of information lost in the projection [see Section 2, specifically Equation (2)], and show that this statistic can capture both the loss of local and of a more global level structure.

For comparison, we evaluated the average number of retained *n*-nearest neighbors for various values of *n* (Jaccard index of the *n* nearest neighbors before and after projection). Small and large *n* represent how well the local relationships (i.e. order of the nearby samples) and global relationship (i.e. order of long-distance samples), respectively, are preserved. The Jaccard index-based metric is expensive to calculate for high-dimensional data (Lee and Verleysen, 2009), while our metric is produced as a side product of t-SNE mapping (see Section 2).

**Fig. 4.**
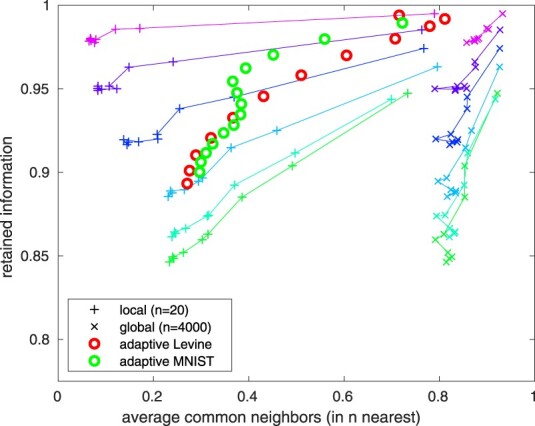
Average number of retained neighbors versus fraction of retained information. The lines indicate 10-D normal problems with 5000 samples, with inherent dimension varying from 2 to 10 (2-D being closest to (1, 1) and 10-D furthest), colors indicating varying perplexity from 1 to 4999 at logarithmically equispaced intervals. The curves with pluses indicate how well local structure is maintained (*n *=* *20) and *x*: s the global structure (*n *=* *4000). The Levine and MNIST datasets are evaluated at their characteristic level of detail (i.e. *n* equal to the perplexity) for various perplexity values

We analyzed synthetic datasets (*k*-dimensional multivariate normal data) with varying inherent dimension of the data embedded in a 10-D space (see Supplementary Material), and evaluated the information loss metric. In these, the structure is random, the complexity being set by the inherent dimension, which is easier to generate and harder to capture than a more realistic data. As shown in Figure 4, the metric correlates well with maintaining both local and global structures. For inherently 2-D problems, a very high degree of information is retained, which is also reflected by the number of retained neighbors at all scales. For higher-dimensional problems, less information is captured by the projection, as expected. We also verified that the metric performs well with practical problems at varying perplexity as evaluated at the characteristic level of detail as shown for the Levine and MNIST datasets.

### 3.4 High-resolution analysis reveals putative chemoresistant and chemosensitive phenotypes in ovarian cancer tumors

To show the benefits of qSNE on a large mass cytometry dataset, we analyzed the levels of 18 proteins in high-grade serous ovarian cancer (HGSOC) patient ascites samples before and after chemotherapy. The proteins were selected to span HGSOC cancer markers (CA-125 and HE4) (Ferraro *et al.*, 2013); epithelial cell markers (MUC1, E-cadherin, EpCAM); immune and inflammatory markers (CD8a, CD45, CD3 and PD1); stromal markers (CD90, CD44 and CD146) and stemness and other markers (CD117, Sox2, CD24, CD133-APC, N-cadherin and CD166-PE). The dataset consists of 173 374 cells, which is impractical to analyze using the traditional t-SNE algorithm. However, a dataset of this scale poses no challenge to qSNE, which generated a full-resolution mapping in ∼2 h 15 min of computation.

**Fig. 5.**
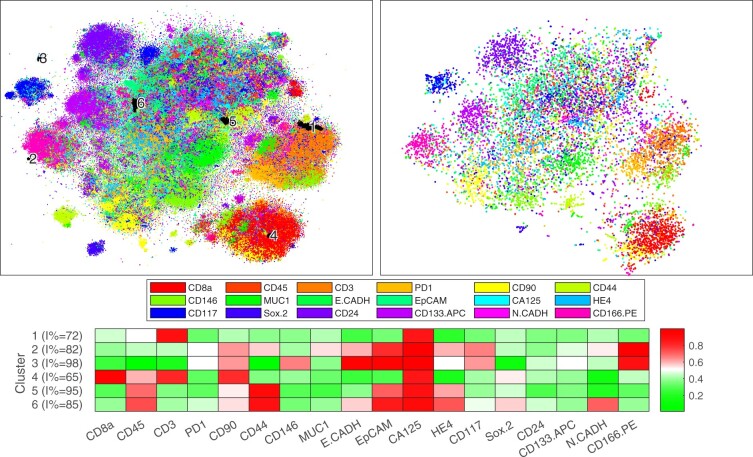
t-SNE mappings for a combined sample before and after chemotherapy for an ovarian cancer patient. Left panel: qSNE (173 374 cells, perplexity 150, Hessian matrix rank 5); and right panel: Rtsne (10 000 cells, perplexity 50). The hue indicates the most prominent marker, as indicated in the legend, and the saturation its level. Black highlighting indicates clusters of interest that are not identifiable in the downsampled analysis (unhighlighted data in Supplementary Fig. S4). The bottom panel shows a heatmap of the average expression of the highlighted clusters. (Color version of this figure is available at *Bioinformatics* online.)

To compare the full-resolution mapping to the traditional subsampling, we acquired a mapping using Rtsne using 10 000 randomly subsampled cells. The mappings are shown in Figure 5 on a similar scale, and a higher fidelity version of the full dataset is shown in Supplementary Figure S4, the color encoding the most prominent marker. The full resolution analysis by qSNE revealed several phenotypic clusters that were not identifiable in the downsampled data. For example, various likely stromal (e.g. CD90, CD44 and CD146 high; yellow color; around the bottom of the plot; see Supplementary Fig. S4) and immune cell clusters (e.g. CD8a, CD45 and CD3 high; red to orange color; bottom right) are visible in both mappings, but unlike in the full-resolution mapping, the cluster substructure is not revealed and the various smaller clusters in between the larger ones appear missing in the lower-resolution analysis. Further analysis of these high-resolution substructures showed that they correlate with whether the cells were subject to the chemotherapy or not (see Fig. 5), which suggests that a full-resolution analysis can aid to distinguish the chemotherapy-sensitive and -resistant phenotypes.

Our expert manually annotated clusters (clusters 1–6) that were dominantly enriched in either of the HGSOC markers CA-125 or HE4 in the full-resolution analysis (see Supplementary Fig. S4) and were rich (>50%) in interval (treated sample) cells (see inset of Supplementary Fig. S4). These clusters are highlighted in Figure 5 along with a heatmap of their average expression. The corresponding cells in the downsampled data were found to be scattered in the several clusters, and consequently not identifiable using the lower resolution analysis alone. Of these, cluster 1 is located near the CD3-positive putative T-cell cluster, while cluster 4 is located within the CD8a positive T-cell cluster, which suggests cancer-interacting immune cell phenotypes. Meanwhile, the other clusters are unlikely to be immune cells, as they are enriched in the epithelial markers, particularly the clusters 2 and 3 located on the opposite side of the visualization. Clusters 2 and 3 are also enriched in the cancer stemness marker CD166, while clusters 5 and 6 are enriched in the leukocytic CD45 marker and in CD44, which has been associated with epithelial ovarian cancer cells with a more favorable treatment response (Sillanpaa *et al.*, 2003). Each of the clusters is specific to the interval (after chemotherapy) sample rather than to the treatment naive sample, featuring significantly more interval cells than expected (*P*-values $<1.6\times 10^{-2}$ in a conditioned binomial test).

## 4 Conclusion

Single-cell measurement technologies generate massive, high-resolution datasets. However, most of the current analysis softwares are not directly able to analyze these data and resort to downsampling, which hinders fully exploiting the high-resolution nature of the data.

We report a novel implementation of the non-parametric dimensionality reduction method t-SNE, called qSNE, which utilizes a quasi-Newton t-SNE optimizer. We show that for many practical problems and parameter settings qSNE allows convergence at quadratic rate, and consequently, an order of magnitude less computation. Importantly, qSNE produces comparable visualizations, despite that it might convergence to a different optimum. In addition, we present a method optimize the input distribution bandwidth, or the perplexity parameter, automatically. This enables the data analyst to focus on only specifying the desired level of detail, and letting the optimizer to deal with parameter tuning. The perplexity tuning also opens up an avenue toward analyzing heteroscedastic data in the complex input space, where no single parameter value can produce satisfactory result. Finally, we proposed a quality metric, which can be obtained as a side product of computing the mapping. This feature is particularly important because at the moment t-SNE visualizations are used without any analysis of the model fitness, which implies that important details of a dataset may remain uncaptured by the model without any sign reported to the analyst. Herein, we propose that a quality metric should be used routinely to assess immediately whether the acquired mapping well represents the original data, which cannot be evaluated using the lower dimensional mapping alone. qSNE is best suited for datasets with 100 000 s samples with 10 to 100 features, such as large mass cytometry data, but we also demonstrated its applicability on single-cell RNA-seq data with 10 000 samples and 18 726 genes.

Our improvements are general enough to be combined with future improvements, such as alternative input and output models (Hinton and Roweis, 2003); tree-based spatial subdivision (van der Maaten, 2014); out of sample extensions, like kernel t-SNE (Gisbrecht *et al.*, 2015) and other interpolation schemes (Linderman *et al.*, 2019; Pezzotti *et al.*, 2020); hyperparameter optimization (Belkina *et al.*, 2019); and GPU parallelization schemes (Chan *et al.*, 2019; Pezzotti *et al.*, 2020). Unlike some of these approaches, we focused here on the exact instead of an approximate problem, as it is application specific whether the approximate schemes allow an analysis at a comparable level of detail.

We demonstrated that such improvements are critical in analyzing large datasets containing complex, infrequent features. Specifically, we demonstrated the utility by analyzing HGSOC mass cytometry data, which was not previously feasible at the attained level of detail. Our analysis revealed cluster of cells which are only identifiable at the higher level of detail, which can aid in developing efficient interventions to overcome HGSOC chemoresistance. qSNE is freely available with documentation.

## Supplementary Material

btaa637_Supplementary_Data
