# Supplementary material for: qSNE: quadratic rate t-SNE optimizer with automatic parameter tuning for large datasets
Source: Bioinformatics. 2020 Jul 14;36(20):5086–92. doi: 10.1093/bioinformatics/btaa637 (PMC7755412; doi:10.1093/bioinformatics/btaa637)
Supplement: btaa637_Supplementary_Data [file btaa637_supplementary_data.zip › supplement.pdf]

Supplementary material for "qSNE: Quadratic rate t-SNE optimizer with automatic parameter tuning for large data sets"

Antti Häkkinen, Juha Koiranen, Julia Casado, Katja Kaipio, Oskari Lehtonen, Eleonora Petrucci, Johanna Hynninen, Sakari Hietanen, Olli Carpén, Luca Pasquini, Mauro Biffoni, Rainer Lehtonen, Sampsa Hautaniemi

Extended methods

The t-SNE algorithm

The t-distributed stochastic neighbor embedding (t-SNE) finds a lower dimensional representation of a data set by optimizing the latent embedding of the samples in a lower dimensional space such that the distribution of local distances between the samples are maintained [S10].

Specifically, t-SNE models the distribution of "neighborness" of each pair of points in the high and low dimensional spaces with probability distributions P and Q, respectively, and seeks to minimize Kullback-Leibler divergence from Q to P, i.e. the information lost when approximating P using Q. In case of normal distribution, this "neighborness" represents a diffusive random walk emanating from an uniformly selected sample first arriving to a specific neighboring node (disregarding the node of origin).

For the samples in the original high-dimensional space, the distribution emanating from sample i using a normal kernel is defined as:

$$p_{i\to j} \mathrel{\dot{\propto}} \frac{\exp\Big(-\frac{1}{2}\,||\,\mathbf{x}_j - \mathbf{x}_i\,||^2\;\sigma_i^{-2}\Big)}{\sum_{j'\neq i}\exp\Big(-\frac{1}{2}\,||\,\mathbf{x}_{j'} - \mathbf{x}_i\,||^2\;\sigma_i^{-2}\Big)}\quad\text{, except for}\quad p_{i\to i} \doteq 0 \tag{S1}$$

where $\sigma_i \in \mathbb{R}_{\geq 0}$ is the bandwidth (standard deviation) on the normal kernel centered on the high-dimensional representation $\mathbf{x}_i \in \mathbb{R}^n$ of the i:th sample (out of m), determining how diffuse the space is around the point (it can be more useful to think this as an approximation to a diffusive field $\sigma(\mathbf{x})$ in the input space which is expanded around the sample $\mathbf{x}_i$ into $\sigma_i = \sigma(\mathbf{x}_i)$). Having a constant bandwidth σ_i around the point $\mathbf{x}_i$ is a good approximation in the vicinity of the point $\mathbf{x}_i$, even when the walk tends closer to the point $\mathbf{x}_j$ provided that the two are close (as $\sigma_j \to \sigma_i$ as $\mathbf{x}_j \to \mathbf{x}_i$ due to how σ_i are selected, see below), but fails when the points are distant (in relation to the other points). If our purpose is to retain the local relationships, this approximation is appropriate. However, in practice such asymmetric neighborness tends to perform poorly when σ_i is held constant over the space, as the neighborness information can only flow from an outlier but not to an outlier, so symmetrized input space distribution is used [S10]. The interpretation of this is to approximate the full diffusive field by interpolating between the unidirectional approximations at the two

endpoints, $\mathbf{x}_i$ and $\mathbf{x}_j$. Specifically, the symmetrized distribution is:

$$P: \; p_{ij} \doteq \frac{1}{2}\Big(p_{i \to j} + p_{j \to i} \Big) \tag{S2}$$

Meanwhile, the distribution in the embedding space is modeled using a t-distribution (Cauchy with $\nu = 1$ degrees of freedom). Other distributions have been considered [S2], but they have not been as successful [S10]. The success of t-distribution over, for instance, Gaussian is attributed to the insensitivity to the neighbors at the tails of the distribution. Specifically, the output distribution is defined as:

$$Q: \; q_{ij} \,\dot{\propto}\, \frac{\Big(1 + ||\,\mathbf{y}_j - \mathbf{y}_i\,||^2 \, \gamma^{-2}\Big)^{-1}}{\sum_{i' \neq j'} \Big(1 + ||\,\mathbf{y}_{j'} - \mathbf{y}_{i'}\,||^2 \, \gamma^{-2}\Big)^{-1}} \quad \text{, except for} \quad q_{ii} \doteq 0 \tag{S3}$$

where $\mathbf{y}_i \in \mathbb{R}^d$ is the low dimensional representation of the i:th sample, the scale $\gamma \in \mathbb{R}_{\geq 0}$ can be taken as $\gamma = 1$ without loss of generality, and which is already naturally symmetric. UMAP can be thought to have similar local similarity distribution with the distances distorted by a power law (i.e. $||\cdot|| \to ||\cdot||^b$ with $b \approx 0.7915$ by default) [S6], which can be considered as inverse distance weighing.

Now, the information lost when using Q to approximate P, the Kullback-Leibler divergence from Q to P and minimized by t-SNE, is given by:

$$C \doteq D_{\mathrm{KL}}(P\,||\,Q) = \sum_{i=1}^{m} \sum_{j=1}^{m} -p_{ij} \, \log \frac{q_{ij}}{p_{ij}} \tag{S4}$$

A critical step much affecting the acquired projection is to choose the bandwidths σ_i in the input space. This is commonly done by selecting the bandwidths by the means of "perplexity", a continuous measure of the number of neighbors:

$$\sigma_i \quad \text{such that} \quad \pi_i \doteq \exp(H(P_i)) = \exp\Big(\sum_{j=1}^{m} -p_{i \to j} \, \log p_{i \to j} \Big) \tag{S5}$$

where $H(P_i)$ is the entropy of the input distribution emanating from the i:th sample and $\pi_i \in [0, m-1]$ represents the chosen perplexity, corresponding to the number of neighbors for a binary neighbor membership distribution ($\exp(H(P_i)) = k$ if $p_{i \to j}$ has k equal nonzeros).

In the original t-SNE implementation, the minimization is implemented through a modified gradient descent procedure. Two hyperparameters are involved in the updates, namely momentum $\mu(t)$ and learning rate $\eta(t)$. Iterates are obtained using:

$$\mathbf{y}_{:}(t{+}1) = \mathbf{y}_{:}(t) \; - \; \underbrace{\eta(t) \; \nabla_{\mathbf{y}_{:}} \, C(\mathbf{y}_{:}(t))}_{\text{gradient term}} \; + \; \underbrace{\mu(t) \, \Big(\mathbf{y}_{:}(t) - \mathbf{y}_{:}(t-1) \Big)}_{\text{momentum term}} \tag{S6}$$

where $\mathbf{y}_{:}(t) = \mathrm{vec}(\,[\,\mathbf{y}_1(t), \cdots, \mathbf{y}_n(t)\,]\,)$. The negative gradient term drives the iterates toward a minimum and the momentum mixes in information from previous iterates. In the original t-SNE implementation, $\eta(t)$ is initially 100 and modified adaptively on subsequent iterations, such that $\mu(t) = 0.5$ for $t < 250$ and $\mu(t) = 0.8$ for $t > 250$. In addition, t-SNE uses a trick called "early exaggeration", where the input similarities are multiplied by a constant value for a specific number of iterations, which essentially increases the effective learning rate $\eta(t)$ during the early part of the optimization process. [S10]

It is common to take the initial guess $\mathbf{y}(0)$ from the most significant components of a (linear) principle component analysis (PCA), and running updates using Equation (S6) for either a fixed number of iterations or until the update magnitude vanishes to a specified tolerance. It suffices to compute the input distribution using Equation (S2) only once, while the output distribution must be updated after each iteration using Equation (S3), as this depends on the current embedding $\mathbf{y}(t)$.

For a practical implementation, the objective gradient must be explicitly evaluated:

$$\nabla_{y_{ik}} C(\mathbf{y}_{:}) = \sum_{s=1}^{m} (p_{is} - q_{is})\, 4\, Z\, q_{is}\, (y_{ik} - y_{sk}) \tag{S7}$$

where $Z \doteq \sum_{i' \neq j'} (1 + ||\, \mathbf{y}_{j'} - \mathbf{y}_{i'}\, ||^2)^{-1}$ is the partition function (normalizing factor) for Q. The gradient was already reported by van der Maaten & Hinton [S10], albeit lacking the exact scaling factor. Differentiating the gradient, gives the Hessian matrix of:

$$\begin{aligned}
\nabla\nabla^{*}_{y_{ik}\, y_{jl}} C(\mathbf{y}_{:}) = &\left(\sum_{s=1}^{m} q_{is}\, 4\, Z\, q_{is}\, (y_{ik} - y_{sk}) \right) \left(\sum_{s=1}^{m} q_{sj}\, 4\, Z\, q_{sj}\, (y_{sl} - y_{jl}) \right) \quad + \\
&\frac{1}{2}\, (p_{ij} - 2\, q_{ij}) \left(4\, Z\, q_{ij}\, (y_{ik} - y_{jk}) \right) \left(4\, Z\, q_{ij}\, (y_{il} - y_{jl}) \right) \quad + \\
\frac{1}{2}\, \delta_{i=j} &\sum_{s=1}^{m} (p_{is} - 2\, q_{is}) \left(4\, Z\, q_{is}\, (y_{ik} - y_{sk}) \right) \left(4\, Z\, q_{sj}\, (y_{sl} - y_{jl}) \right) \quad + \\
\delta_{k=l} &\left(-\, (p_{ij} - q_{ij})\, 4\, Z\, q_{ij} + \delta_{i=j} \sum_{s=1}^{m} (p_{is} - q_{is})\, 4\, Z\, q_{is} \right)
\end{aligned} \tag{S8}$$

where δ_x is an indicator, i.e. unity if x is true and zero otherwise. In general, the second term is full, and so is the Hessian matrix, while the others are lower rank or damping structures. However, the Hessian matrix is used neither in the original nor in our implementation, but presented for comparison.

Fast quasi-Newton optimization

The original gradient descent scheme only allows linear convergence, which can be prohibitively slow on large data sets, as the objective complexity increases exponentially. Algorithms that employ a quadratic approximation of the objective (instead of the linear approximation of the gradient descent) permit quadratic convergence. Unfortunately, evaluating the Hessian matrix directly is too expensive (complexity of $\mathcal{O}(m^2)$), so we decided to employ the limited-memory Broyden-Fletcher-Goldfarb-Shanno method (L-BFGS) [S5]. This combines potentially quadratic convergence with low computational overhead as the full Hessian matrix need not to be evaluated but a low rank approximation to it is used. Typically the rank of the Hessian matrix approximation can be some small constant, such that quadratic complexity per iteration is avoided but that the approximation retains much of the power of the Hessian operator, and consequently, performance of a Newton optimizer.

For a Newton (quadratic) optimizer, the next iterate is obtained as follows:

$$\mathbf{y}_{:}(t+1) = \mathbf{y}_{:}(t) \; - \; \underbrace{\alpha(t)\; \mathbf{H}(t)^{-1}\; \nabla_{\mathbf{y}_{:}} C(\mathbf{y}_{:}(t))}_{\text{gradient term}} \tag{S9}$$

where $a(t)$ is the step length, $\mathbf{H}(t)^{-1}$ the inverse Hessian matrix of C with respect to $\mathbf{y}_{:}$ at $\mathbf{y}_{:}(t)$. Note that the learning rate is now automatically set to the (locally optional) value of the inverse Hessian matrix, instead of adhering to a fixed rate, and that the rate can vary to different directions. For a quasi-Newton method, like the L-BFGS, an approximation is substituted for the inverse Hessian matrix.

Let $\tilde{\mathbf{H}}_t^{-1}$ denote the approximate inverse Hessian matrix at the t:th iterate. In L-BFGS, the approximation is updated according to [S5]:

$$\tilde{\mathbf{H}}_{t+1}^{-1} = \mathbf{V}_t^* \, \tilde{\mathbf{H}}_t^{-1} \, \mathbf{V}_t + \rho_t \, \mathbf{s}_t^* \, \mathbf{s}_t \tag{S10}$$

where:

$$\begin{aligned} \rho_t &\doteq \frac{1}{\boldsymbol{\gamma}_t^* \, \mathbf{s}_t} & V_t &\doteq \mathbf{I} - \rho_t \, \boldsymbol{\gamma}_t \, \mathbf{s}_t^* \\ \mathbf{s}_t &\doteq \mathbf{y}_{:}(t+1) - \mathbf{y}_{:}(t) & \boldsymbol{\gamma}_t &\doteq \nabla_{\mathbf{y}_{:}} C(\mathbf{y}_{:}(t+1)) - \nabla_{\mathbf{y}_{:}} C(\mathbf{y}_{:}(t)) \end{aligned} \tag{S11}$$

which essentially applies a rank-1 update to correct the Hessian matrix (not the inverse) approximation from $\tilde{\mathbf{H}}_t$ toward the true Hessian matrix $\mathbf{H}(t+1)$. Moreover, the approximation allows computing the search direction $-\tilde{\mathbf{H}}_t^{-1} \, \nabla_{\mathbf{y}_{:}} C(\mathbf{y}_{:}(t))$ efficiently from the latest r pairs of $\mathbf{s}_t$, $\boldsymbol{\gamma}_t$ without the need to store a full inverse Hessian matrix approximation in memory, as follows:

(SA1)

Data: Current gradient $\nabla_{\mathbf{y}_{:}} C(\mathbf{y}_{:}(t))$, previous updates $\mathbf{s}_t$ and $\boldsymbol{\gamma}_t$, and ρ_t as above
Result: Search direction $\mathbf{p} \doteq -\tilde{\mathbf{H}}_t^{-1} \, \nabla_{\mathbf{y}_{:}} C(\mathbf{y}_{:}(t))$
$\mathbf{q} \leftarrow \nabla_{\mathbf{y}_{:}} C(\mathbf{y}_{:}(t))$
for $i = t-1, t-2, \ldots, t-r$ **do**
  $\alpha_i \leftarrow \rho_i \, \mathbf{s}_i^T \, \mathbf{q}$
  $\mathbf{q} \leftarrow \mathbf{q} - \alpha_i \, \boldsymbol{\gamma}_i$
end
$\mathbf{p} \leftarrow -\tilde{\mathbf{H}}_0^{-1} q$
for $i = t-r, t-r+1, \ldots, t-1$ **do**
  $\beta_i \leftarrow \rho_i \, \boldsymbol{\gamma}_i^T \, \mathbf{p}$
  $\mathbf{p} \leftarrow \mathbf{p} - (\alpha_i - \beta_i) \, \mathbf{s}_i$
end

where $\tilde{\mathbf{H}}_0^{-1}$ is some initial inverse Hessian matrix approximation, often, and in our implementation, an identity matrix, in the lack of better insight for efficient computation. From this, it is apparent that the scheme requires only $\mathcal{O}(r\,k)$ memory and time, where r is the approximation rank, $k = m\,d$ is the Hessian matrix dimension, m is the number of samples, and d is the dimension of the low-dimensional space; and that this procedure reduces to gradient descent for $r = 0$.

To maintain stability, a line search procedure is required as the quadratic approximation may become poor far away from the point of approximation $\mathbf{y}_{:}(t)$. The line search procedure reduces the step length α until a sufficient decrease in the objective function is obtained, that is, the Armijo condition is satisfied. We note that the line search usually proceeds with a full step length, unless the optimizer is already very close to the optimum (in which case the solver can be stopped), and is thus very cheap (involving $\mathcal{O}(1)$ objective evaluations). A backtracking line search can be implemented as:

Data: Point of approximation $\mathbf{y}_{:}(t)$ and the search direction $\mathbf{p}$
Result: Adequate step length α
$\alpha \leftarrow 1$
while $C(\mathbf{y}_{:}(t) + \alpha\,\mathbf{p}) > C(\mathbf{y}_{:}(t)) + c_1\,\alpha\,\mathbf{p}^*\ \nabla_{\mathbf{y}_{:}} C(\mathbf{y}_{:}(t))$ **do**
  $\alpha \leftarrow c_2\ \alpha$
end (SA2)

where $c_1 \in (0,1)$, $c_2 \in (0,1)$ are the search parameters, c_1 relating to the quality of the approximation and c_2 being a step size for the search. For our implementation $c_1 = c_2 = 0.5$ is used following the previous literature [S7].

Since the line search does not guarantee that the curvature condition $\boldsymbol{\gamma}_t^*\,\mathbf{s}_t > 0$ holds for the update, we also implement a damping procedure to maintain the positive definiteness of the Hessian matrix approximation, guaranteeing that the search direction is always a descent direction [S7]. The L-BFGS update formula is modified by replacing $\boldsymbol{\gamma}_t$ with $\tilde{\boldsymbol{\gamma}}_t$ as follows:

$$\tilde{\boldsymbol{\gamma}}_t = \theta_t\,\boldsymbol{\gamma}_t + (1-\theta_t)\ \tilde{\mathbf{H}}_t^{-1}\,\mathbf{s}_t \tag{S12}$$

where

$$\theta_k = \begin{cases} 1 & \text{if } \boldsymbol{\gamma}_t^*\,\mathbf{s}_t \geq \sigma\,\mathbf{s}_t^*\ \tilde{\mathbf{H}}_t^{-1}\,\mathbf{s}_t \\ \Big((1-\sigma)\,\mathbf{s}_t^*\,\tilde{\mathbf{H}}_t^{-1}\,\mathbf{s}_t\Big)\ /\ \Big(\mathbf{s}_t^*\,\tilde{\mathbf{H}}^{-1}\,\mathbf{s}_t - \boldsymbol{\gamma}_t^*\,\mathbf{s}_t\Big) & \text{otherwise} \end{cases} \tag{S13}$$

where $\sigma \in (0,1)$ is a suitable damping parameter. In our implementation $\sigma = 0.2$ is used following previous literature [S7]. Again $\tilde{\mathbf{H}}_t^{-1}\,\mathbf{s}_t$ can be computed without explicitly forming $\tilde{\mathbf{H}}_t^{-1}$.

Finally, we note that L-BFGS is a generic procedure, and can be combined with other t-SNE improvements, such as a tree approximations for computing the gradient [S9]. We do not find these advantageous for high-dimensional data (as they are $\mathcal{O}(2^n)$, they might be practical for $n \ll 10$, but not for $n > 20$), and their approximations produce an approximate solution to the t-SNE problem, whereas the L-BFGS approximation produces an exact solution through an approximate (suboptimal) path. Also, a momentum term can be included post-hoc in the update as is with gradient descent.

Automatic perplexity selection

For practical problems, most of the perplexity values are not very interesting: a change in the perplexity value might not result in a significant change in the embedding. To locate the interesting perplexity values, we quantify how much the problem changes as a function of changing the perplexity.

The neighborhood entropy $H(P_i)$ is a monotonic increasing curve from 0 to $\log(m-1)$ as the bandwidth σ_i varies from 0 to ∞. Assuming some local dispersion of clustering σ_i^*, when $\sigma_i \ll \sigma_i^*$ or $\sigma_i \gg \sigma_i^*$, $\nabla_\sigma H(P_i) \approx 0$, as the neighborhood structure remains insensitive to the bandwidth, while for $\sigma_i \approx \sigma_i^*$, $\nabla_\sigma H(P_i) \gg 0$. This works even in the presence of multiple local dispersion factors, as a scale only contributes to the entropy gradient when it is sensitive. In practice, this results in a staircase-like figure (see e.g. Figure 3).

To locate these interesting perplexity values, optima of this functional can be sought. The user must select the interesting level of detail, as often there are higher and lower level features, and consequently, multiple optima. Given a perplexity range, an optimum can be located using sectioning as follows:

Data: Perplexity interval $[\pi_{\min}, \pi_{\max}]$
Result: Optimal bandwidth σ_i^*
Choose lower bound $\sigma_a \leftarrow \sigma_i \,|_{\pi_i=\pi_{\min}}$ using Equation (S5)
Choose upper bound $\sigma_d \leftarrow \sigma_i \,|_{\pi_i=\pi_{\max}}$ using Equation (S5)
$\sigma_b \leftarrow \sigma_b - (\sigma_b - \sigma_a)\,\phi^{-1}$
$\sigma_c \leftarrow \sigma_a + (\sigma_b - \sigma_a)\,\phi^{-1}$
while $\sigma_a \leq \sigma_b \wedge \sigma_c \leq \sigma_d$ **do**
  if $\nabla_\sigma H(P_i)\,|_{\sigma_i=\sigma_b} < \nabla_\sigma H(P_i)\,|_{\sigma_i=\sigma_c}$ **then** (SA3)
    $\sigma_a \leftarrow \sigma_b$
  else
    $\sigma_d \leftarrow \sigma_c$
  end
  $\sigma_b \leftarrow \sigma_b - (\sigma_b - \sigma_a)\,\phi^{-1}$
  $\sigma_c \leftarrow \sigma_a + (\sigma_b - \sigma_a)\,\phi^{-1}$
end
$\sigma^* = \frac{\sigma_a+\sigma_d}{2}$

where $\phi = \frac{1+\sqrt{5}}{2}$ is the golden ratio.

Quality of an acquired mapping

After acquiring a lower dimensional mapping of the data, it is useful to assess how well this representation captures the features of the original data set for the purposes of interpretation. For inherently high-dimensional data sets, we cannot expect a 2-D mapping to capture the underlying structure, whereas if the data lies on a low-dimensional manifold in a high-dimensional space, the mapping is expected to well represent the original data set.

However, quantitative analysis is often not performed, despite that an information-theoretic statistic is readily available as a side product of acquiring the t-SNE mapping. For any mapping in the t-SNE framework, the source entropy $H(P)$ represents the average number of bits needed to encode a sample of the original data, while the Kullback-Leibler information divergence between the source and destination distributions $D_{\text{KL}}(P\,||\,Q)$ is the average number of extra bits needed if the output model is used encode the samples instead. These are readily available during the optimization, and can be evaluated once the optimal mapping has been obtained.

To quantify the quality of the mapping, we propose the following normalized statistic:

$$q \doteq 1 - \frac{H(P^*)}{H(P^*) + D_{\text{KL}}(P^*\,||\,\hat{Q})} = 1 - \frac{\sum_{i=1}^{m} H(P_i^*)}{\sum_{i=1}^{m} H(P_i^*) + D_{\text{KL}}(P_i^*\,||\,\hat{Q}_i)} \tag{S14}$$

where P_i^* is the distribution around the i:th sample for its optimal bandwidth and $\hat{Q}_i$ is the optimal embedding distribution. This quantity has the following rationale: $H(P^*)$ quantifies the bits needed to represent the samples in the original space, while the cross-entropy $H(P^*) + D_{\text{KL}}(P^*\,||\,\hat{Q})$ represents the number of bits needed to encode the data using the lower-dimensional model, so their ratio would be the fraction of samples you can encode in the same space with the output model. As expected, q is zero for one-to-one

correspondence between the source and destination distributions ($D_{pq} = 0$), and unity if all the information is lost ($D_{pq} = \infty$).

Implementation and parameters

The full procedure can be outlined as:

Data: High dimensional input data $\mathbf{x}_1, \ldots, \mathbf{x}_m$, perplexity range $[\pi_{\min}, \pi_{\max}]$, and an initial embedding $\mathbf{y}_1, \ldots, \mathbf{y}_m$ (e.g. the scores on the first k principal components)

Result: Final embedding $\mathbf{y}_i$, quality q

```
// Optimize perplexity
```

for $i = 1, \ldots, m$ **do**

  Optimize perplexity around sample i, giving σ_i^*, using Algorithm (SA3)

  Compute the corresponding perplexity π_i^* and entropy $H(P_i^*)$ using Equation (S5)

end

```
// Optimize embedding
```

for $t = 1, \ldots, \infty$ **do**

```
// Compute Newton update
```

  Use Equation (S11) to compute $\mathbf{s}_t$ and $\boldsymbol{\gamma}_t$. It is convenient to keep the r last iterates $\mathbf{y}_:(t-r), \ldots, \mathbf{y}_:(t-1)$ and $\nabla_{\mathbf{y}} C(\mathbf{y}_:(t-r)), \ldots, \nabla_{\mathbf{y}_:} C(\mathbf{y}_:(t-1))$ in a ring buffer and compute $\mathbf{s}_t$ and $\boldsymbol{\gamma}_t$ as needed. (SA4)

  Damp $\boldsymbol{\gamma}$ giving $\tilde{\boldsymbol{\gamma}}$ using Equation (S12)

  Find the Newton search direction $\mathbf{p}$ using Algorithm (SA1)

  Line search a safe Newton step size α using Algorithm (SA2)

  Update embedding $\mathbf{y}_: \leftarrow \mathbf{y}_: - \alpha\,\mathbf{p}$ using the Newton rule from Equation (S9)

```
// Evaluate stopping condition
```

  Compute objective $D_{\mathrm{KL}}(P^* \,||\, Q)$ using Equation (S4)

  Break loop if the change in $D_{\mathrm{KL}}(P^* \,||\, Q)$ is small (no objective progress) or $||\,\mathbf{p}\,||^2$ is small (no embedding progress)

end

```
// Evaluate quality
```

Let $\hat{Q} \leftarrow Q$ is the optimal embedded neighbor distribution

Compute $D_{\mathrm{KL}}(P^* \,||\, \hat{Q})$ using Equation (S4)

Compute q using Equation (S14)

Our implementation is available at `https://bitbucket.org/anthakki/qsne/` under the simplified BSD license. It comes with a standalone command line program and MATLAB and R interfaces, all of which use the same underlying implementation, and thus give equal results and have comparable performance (besides I/O overhead). The core implementation is implemented in C, and requires `lapack` [S1] for performing the initial PCA. The program supports parallelization through POSIX threads and Intel SIMD (SSE and AVX) for parallelization. AVX SIMD can compute up to 16 FLOPS per instruction (FMA).

The t-SNE mapping for the Fisher iris data set can with or without perplexity tuning and the quadratic algorithm be obtained using the command line program as (given as Bourne shell commands):

```
# download the Fisher iris data from http://archive.ics.uci.edu/ml/datasets/Iris
./test/get_iris.sh >iris.full.txt
# obtain t-SNE mapping using perplexity tuning and the quadratic algorithm
qsne -d2 -p15:60 -m10 -o iris.2d-qsne.txt iris.full.txt
# obtain t-SNE mapping using the original algorithm
qsne -d2 -p30 -C -o iris.2d-tsne.txt iris.full.txt
```

where `iris.full.txt` is the input file with rows corresponding to samples and the column variables (here 4-D), `iris.2d-qsne.txt` is the output file containing the 2-D embedding (`-d2`) with perplexity tuned in range $[15, 60]$ (`-p15:60`) optimized with the quadratic algorithm with an approximate Hessian matrix rank of 10 (`-m10`), and `iris.2d-tsne.txt` is the corresponding embedding using the original algorithm [S10] with a fixed perplexity of 30. Full documentation concerning the parameters and the MATLAB and R interfaces is available at `https://bitbucket.org/anthakki/qsne/`.

For comparison, we used Rtsne version 0.15 (`https://github.com/jkrijthe/Rtsne`), which uses a C++ implementation by van der Maaten [S9] (`https://github.com/lvdmaaten/bhtsne`). This package is also available through CRAN (`https://cran.r-project.org/web/packages/Rtsne/`). The same PCA initialization as for qSNE was used, instead of the default (random normal). Default parameters were used unless otherwise mentioned.

All tests were run on an Intel Xeon cluster featuring AVX2 with up to 46×2.4 GHz cores and 242 GB of RAM and running Ubuntu 18.04, gcc 7.4.0, LAPACK 3.7.1, and R 3.6.2. All test were run using a single thread unless otherwise mentioned, and CPU time (time the computer is executing the specific process, as opposed to other tasks or waiting for I/O) instead of wall clock time was quantified.

Data curation

The HGSOC ascites samples were collected and analyzed at different time points during the patient treatment, first at the time of diagnosis and second after one cycle of chemotherapy. Cells were isolated from the sample and contaminating blood cells were discarded. The suspension of isolated cells was prepared following a standard CyTOF protocol with EQTM Four Element Calibration beads (Fluidigm). The data were acquired with a CyTOF 1 (DVS Sciences/Fluidigm) instrument and software version 5.1.648. The CyTOF software was used for signal normalization and FlowJo (FlowJo LLC) was used for bead-normalization and gating of single-viable cells (DNA markers $^{191/193}$Iridium and ^{103}Rhodium). Only markers identifying the relevant cell types were kept for analysis: CA-125, HE4, MUC1, E-cadherin, EpCAM, CD8a, CD45, CD3, PD1, CD90, CD44, CD146, CD117, Sox2, CD24, CD133, N-cadherin, and CD166. After gating the viable cells, the primary sample featured 98,521 events and the interval sample had 128,388. To obtain a balanced data set we randomly sampled 50% of the cells from each sample, to a total of 173,374 cells. As each sample was acquired separately, each variable in both the primary and interval samples were normalized by centering the mean and scaling to unit variance before combining them into the final data set. From this data set we uniformly subsampled 10,000 cells, a typical quantity when working with Rtsne, by further subsampling 5,000 primary and interval cells.

Ethics approval and consent to participate

The ovarian cancer data were collected at Turku University Hospital. The study and the use of all clinical material have been approved by The Ethics Committee of the Hospital District of Southwest Finland (ETMK) under decision number EMTK: 145/1801/2015. All the patients participating in the study gave written informed consent.

Multivariate normal simulations

Hierarchical multivariate normal data were used to assess the ability of the perplexity optimization to identify the distinct hierarchical levels. For this, 5 supercluster centroids were generated from a 10-dimensional standard multivariate distribution (sd of 1), each of which were used to generate 5 subcluster centroids from a 10-D multivariate normal with sd of 0.25, which were uniformly used to generate the 10-D samples with sd of 0.01. Specifically:

$$\begin{aligned}
\boldsymbol{\mu}_i^{(1)} &\sim \mathcal{N}(\boldsymbol{\mu}^{(0)}, \sigma_1^2\,\mathbf{I}_d) && \text{for } i \in \{1,\ldots,n_1\} \\
\boldsymbol{\mu}_{i,j}^{(2)} &\sim \mathcal{N}(\boldsymbol{\mu}_i^{(1)}, \sigma_2^2\,\mathbf{I}_d) && \text{for } i \in \{1,\ldots,n_1\}, j \in \{1,\ldots,n_2\} \\
(i_k, j_k) &\sim \mathcal{U}(\{1,\ldots,n_1\} \times \{1,\ldots,n_2\}) && \text{for } k \in \{1,\ldots,n_3\} \\
\mathbf{x}_k &\sim \mathcal{N}(\boldsymbol{\mu}_{i_k,j_k}^{(2)}, \sigma_3^2\,\mathbf{I}_d) && \text{for } k \in \{1,\ldots,n_3\}
\end{aligned} \tag{S15}$$

where $\boldsymbol{\mu}^{(0)}$ is the d-dimensional global centroid, $\boldsymbol{\mu}_i^{(1)}$ represent the supercluster centroids, $\boldsymbol{\mu}_{i,j}^{(2)}$ the subcluster centroids, (i_k, j_k) the cluster labels, and $\mathbf{x}_k$ the samples. Further, σ_1, σ_2, σ_3 are the supercluster, subcluster, and sample standard deviations, respectively, and n_1, n_2, n_3 are their counts. Following the above, we have $d = 10$, $\boldsymbol{\mu}^{(0)} = \mathbf{0}_d$, $\sigma_1 = 1$, $\sigma_2 = 0.25$, $\sigma_3 = 0.01$, $n_1 = 5$, $n_2 = 5$, and $n_3 = 1{,}000$. Here, $\mathbf{0}_d$ is a d-dimensional zero vector, $\mathbf{I}_d$ is a d-dimensional identity matrix, $\mathcal{N}(\boldsymbol{\mu}, \boldsymbol{\Sigma})$ denotes a normal distribution with a mean of $\boldsymbol{\mu}$ and covariance of $\boldsymbol{\Sigma}$, and $\mathcal{U}(\Omega)$ denotes an uniform distribution on the set Ω.

Multivariate normal data with varying inherent dimension were generated to assess the ability of the quality metric to capture the loss of information when trying to represent high-dimensional data using a lower-dimensional mapping. For this, were generated multivariate normal samples on a lower dimensional inherent dimension d_i, generate a random higher dimensional basis on the d_e-D ambient space into which these data are embedded into, and transform the data onto that basis. Specifically:

$$\begin{aligned}
\mathbf{y}_k &\sim \mathcal{N}(\mathbf{0}_{d_i}, \mathbf{I}_{d_i}) && \text{for } k \in \{1,\ldots,n\} \\
\mathbf{z}_j &\sim \mathcal{N}(\mathbf{0}_{d_e}, \mathbf{I}_{d_e}) && \text{for } j \in \{1,\ldots,d_i\} \\
\mathbf{B}_{:,j} &= \mathbf{z}_j \,/\, ||\, \mathbf{z}_j \,||_2 && \text{for } j \in \{1,\ldots,d_i\} \\
\mathbf{x}_k &= \mathbf{B}\,\mathbf{y}_k && \text{for } k \in \{1,\ldots,n\}
\end{aligned} \tag{S16}$$

where $\mathbf{y}_k$ represent the samples in their inherent low-dimensional (d_i-D) space, $\mathbf{z}_k$ are the basis directions, $\mathbf{B}$ is a unitary basis, $\mathbf{x}_k$ are represent the samples embedded into a higher dimensional (d_e-D) space, and n is the number of samples. By construction, the d_i basis vectors of $\mathbf{B}$ lie uniformly distributed on a d_e-D hypersphere, which implies that the direction is uniform random but the transformation does not scale the data. In our simulations, the ambient dimension is $d_e = 10$ and the inherent dimension d_i varies in

$\{2, \ldots, 10\}$, and $n = 5{,}000$ samples were generated. Note that the low-dimensional representation $\mathbf{y}_k$ can be exactly recovered using principal component analysis with d_i principal vectors, implying that in the case $d_i = 2$ there also need not to be information loss between the original data and a 2-D t-SNE mapping, while for $d_e > 2$ this almost surely occurs.

Applying qSNE on single-cell RNA-seq data

We also tested the applicability of qSNE on a simulated single-cell RNA-seq data set. This differs from the mass cytometry data in that the number of the features is typically much higher, while the number of samples (cells) is still considerable.

Specifically, we used Splatter [S11] to generate a data set with 18,726 genes and 10,000 cells. The parameters were inferred from the Tung data set [S8], which is a single cell RNA-seq data set of induced pluripotent stem cells from three HapMap individuals [S8, S11]. The frequency of each individual being was set to 0.6, 0.25, and 0.15, respectively, as used by Zappia et al. [S11].

The corresponding convergence plot is shown in Figure S6 for various parameters and the t-SNE embedding with perplexity 30 is shown in Figure S5. The results confirm that qSNE retains a faster convergence rate and can compute a comparable visualization in much fewer iterations also on datas ets with a much higher number of features, such as single-cell RNA-seq data. The higher-dimensional single-cell data requires a higher rank of the Hessian matrix approximation than the Levine and MNIST data sets for truly quadratic convergence (cf. Figure S6), but still the other improvements apply. The computation took $\sim$2.6 min and $\sim$9 min at Hessian matrix rank of 11 and 100 for qSNE, and $\sim$10 min in linear t-SNE mode.

Availability of data and materials

The data sets analyzed and/or generated during the current study are available at `https://github.com/lmweber/benchmark-data-Levine-32-dim` (Levine), `http://yann.lecun.com/exdb/mnist/` (MNIST), or are available from the corresponding author on reasonable request.

The scRNA-seq data set was generated using Splatter version 1.13.0 [S11], which is available at `https://github.com/Oshlack/splatter`, and the model parameters were inferred from the Tung data set [S8], avalable at `https://github.com/jdblischak/singleCellSeq`.

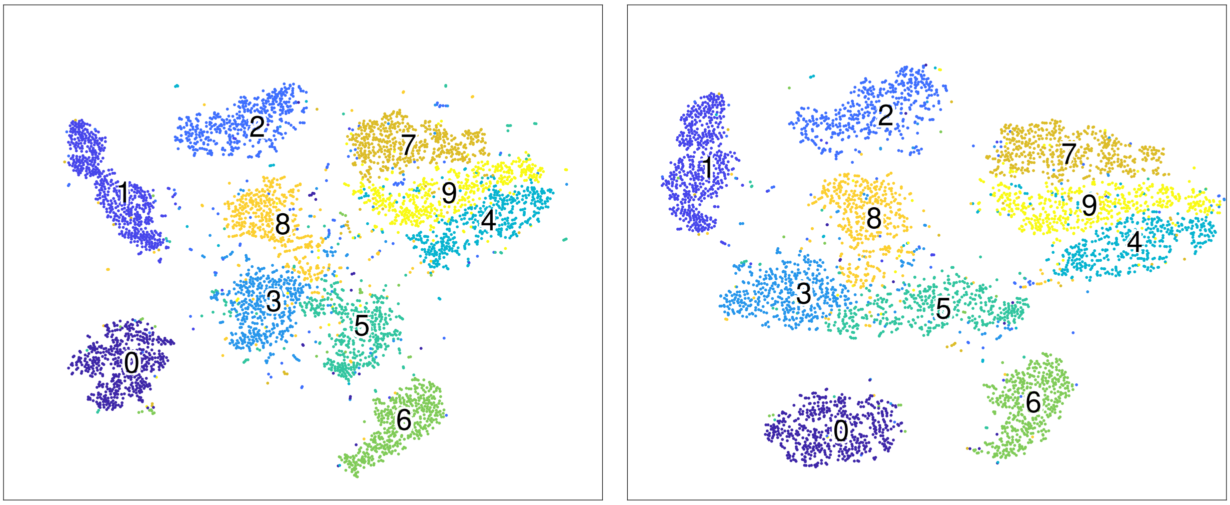

Figure S1: MNIST hand drawn digits data mapped into 2-D. Left panel: qSNE with rank-11 Hessian matrix approximation after 1,092 iterations; and right panel: t-SNE after 9,950 iterations (cf. Figure 1). The data sets were randomly downsampled to 5,000 samples and perplexity was set to 40 in both cases.

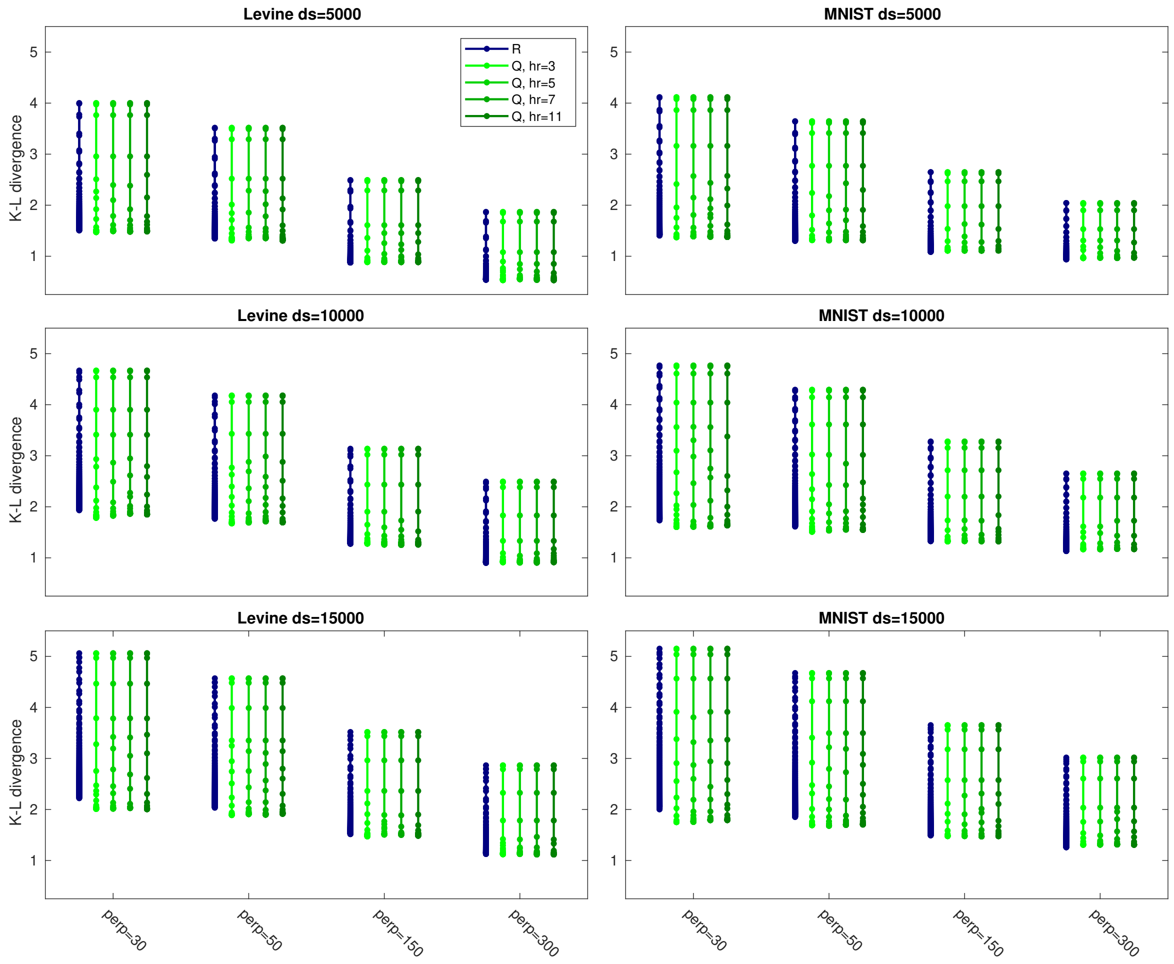

Figure S2: Progress for various downsampling factors, perplexity settings, and Hessian matrix approximation ranks. Left panels: progress, as quantified by the objective value, for both the Levine [S4] data set downsampled to 5,000, 10,000, 15,000 samples at various perplexities for both our quadratic implementation (Q) with various ranks of Hessian matrix approximation (hr) and for a linear Rtsne implementation (R). The dots indicate the objective value after each iteration. Right panels: the corresponding data for the MNIST [S3] data set.

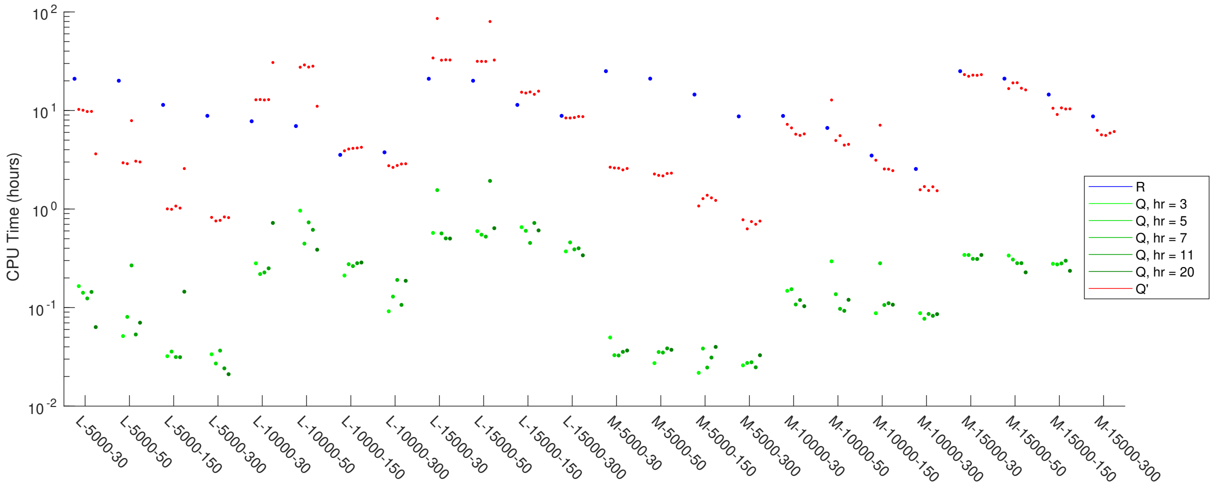

Figure S3: CPU run time. The downsampling factor, perplexity, and the rank of the Hessian matrix approximation (hr) is varied for both the Levine (L) [S4] and MNIST (M) [S3] data sets (indicated as data set-downsampling factor-perplexity on the horizontal axis). R denotes the linear Rtsne implementation, Q our quadratic qSNE implementation, and Q' the predicted qSNE time assuming an equal number of iterations to the Rtsne case.

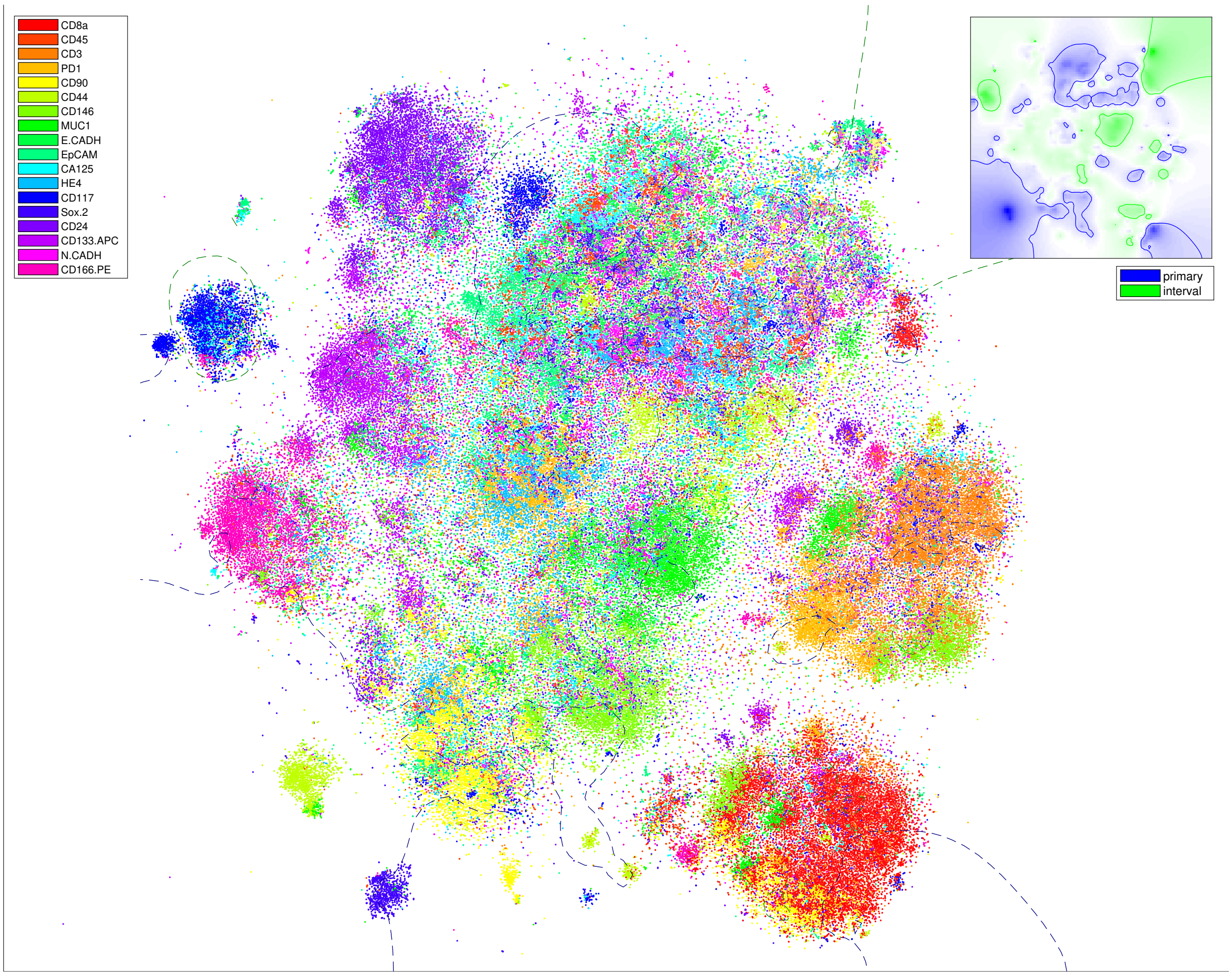

Figure S4: t-SNE mapping for a combined sample before and after chemotherapy for an ovarian cancer patient. The hue indicates the most prominent marker, as indicated in the legend, and the saturation its level. The dashed lines outline regions where cells from either before (primary) or after (interval) chemotherapy are dominant, as indicated by the kernel density estimated likelihood ratio histogram show in the top right corner.

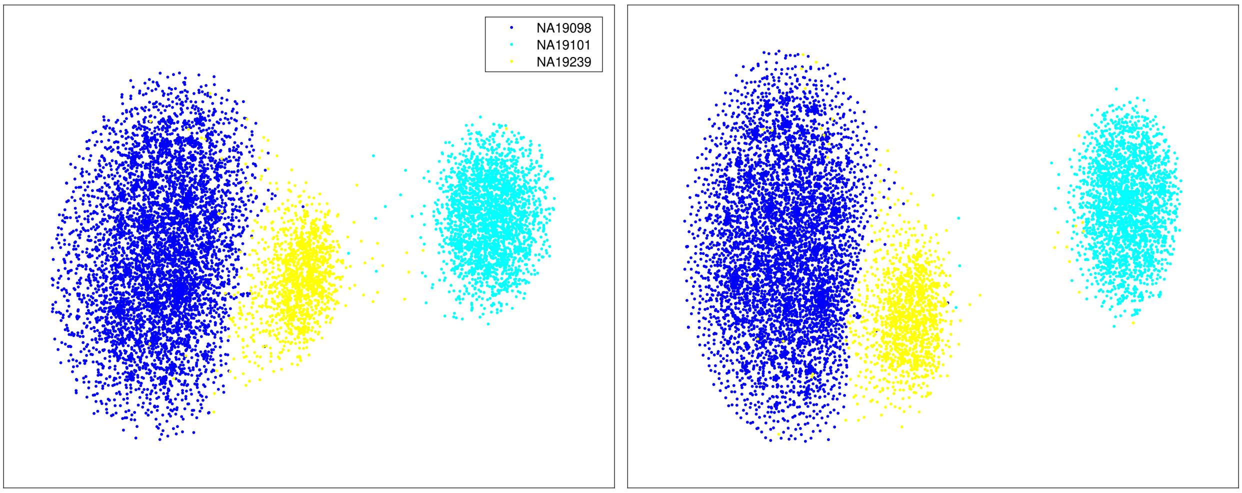

Figure S5: Splatter [S11] generated single-cell RNA-seq data mapped into 2-D. Left panel: qSNE with rank-100 Hessian matrix approximation after 200 iterations; and right panel: t-SNE after 2,650 iterations. Perplexity was set to 30 in both cases.

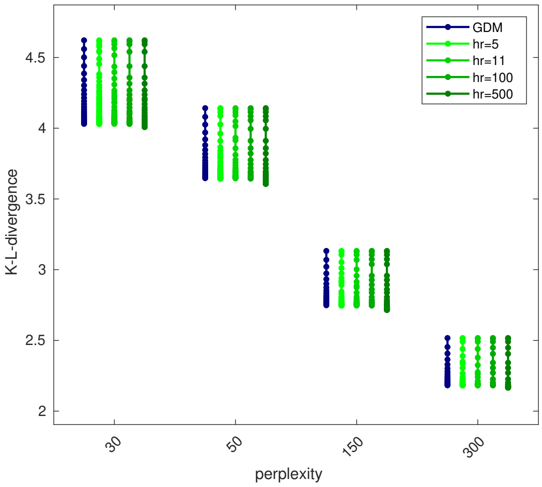

Figure S6: Progress for various downsampling factors, perplexity settings, and Hessian matrix approximation ranks for the Splatter [S11] single-cell RNA-seq data. Progress, as quantified by the objective value at various perplexities for both our quadratic implementation with various ranks of Hessian matrix approximation (hr) and for a linear Rtsne implementation (GDM). The dots indicate the objective value after each iteration.

References

[S1] Anderson, E., Bai, Z., Dongarra, J., Greenbaum, A., McKenney, A., Du Croz, J., Hammarling, S., Demmel, J., Bischof, C., and D., S. (1990). LAPACK: a portable linear algebra library for high-performance computers. In *'90 Proceedings of the 1990 ACM/IEEE conference on Supercomputing*, pages 2–11.

[S2] Hinton, G. E. and Roweis, S. T. (2003). Stochastic neighbor embedding. In S. Becker, S. Thrun, and K. Obermayer, editors, *Advances in Neural Information Processing Systems*, volume 15, pages 857–864. MIT Press.

[S3] Lecun, Y., Bottou, L., Bengio, Y., and Haffner, P. (1998). Gradient-based learning applied to document recognition. *Proc. IEEE*, **86**(11), 2278–2324.

[S4] Levine, J. H., Simonds, E. F., Bendall, S. C., Davis, K. L., Amir, E. D., Tadmor, M. D., Litvin, O., Fienberg, H. G., Jager, A., Zunder, E. R., Finck, R., Gedman, A. L., Radtke, I., Downing, J. R., Pe'er, D., and Nolan, G. P. (2015). Data-driven phenotypic dissection of AML reveals progenitor-like cells that correlate with prognosis. *Cell*, **162**(1), 184–197.

[S5] Liu, D. C. and Nocedal, J. (1989). On the limited memory BFGS method for large scale optimization. *Math. Programming*, **45**(1–3), 503–528.

[S6] McInnes, L., Healy, J., and Melville, J. (2018). UMAP: Uniform manifold approximation and projection for dimension reduction. *arXiv*, **arXiv:1802.03426v2**.

[S7] Nocedal, J. and Wright, S. J. (1999). *Numerical optimization*. Springer, New York, NY.

[S8] Tung, P.-Y., Blischak, J. D., Hsiao, C. J., Knowles, D. A., Burnett, J. E., Pritchard, J. K., and Gilad, Y. (2017). Batch effects and the effective design of single-cell gene expression studies. *Sci. Rep.*, **7**(1), 39921.

[S9] van der Maaten, L. (2014). Accelerating t-SNE using tree-based algorithms. *J. Mach. Learn. Res.*, **15**(Oct), 3221–3245.

[S10] van der Maaten, L. and Hinton, G. (2008). Visualizing data using t-SNE. *J. Mach. Learn. Res.*, **9**, 2579–2605.

[S11] Zappia, L., Phipson, B., and Oshlack, A. (2017). Splatter: simulation of single-cell RNA sequencing data. *Genome Biol.*, **18**(1), 174.
